# Understanding Family Caregivers’ Experiences With Live-in Migrant Care Workers in Dementia Care: Challenges and Perspectives From a Qualitative Study in Taiwan

**DOI:** 10.1177/00469580251355826

**Published:** 2025-07-08

**Authors:** Chia-Ming Yen

**Affiliations:** 1 National Health Research Institutes, National Center of Geriatrics and Welfare Research, Yunlin County, R.O.C Taiwan; 2 Ph.D Program for Aging, China Medical University, Taichung, R.O.C. Taiwan

**Keywords:** home-based dementia care, family caregivers, migrant care workers, qualitative study, Taiwan

## Abstract

Live-in migrant care workers constitute a vital labor force in home-based eldercare in Taiwan; where demographic changes have heightened the demand for such assistant. Despite this, qualitative research exploring the experiences of family caregivers who employ these workers for relatives with dementia remains scarce in the Taiwanese context. This qualitative study aimed to investigate the motivations behind families’ decisions to hire migrant workers for home-based dementia care within Taiwan, as well as to assess the associated benefits and challenges they encounter. In-depth interviews were conducted with 4 family caregivers, aged between 52 and 63 years, who had hired live-in migrant care workers between April and August 2022. The transcripts from these interviews were analyzed thematically to derive insights from the findings. The results revealed that family caregivers in Taiwan opted to hire migrant workers for dementia care following a thorough evaluation of their personal circumstances and available resources. Live-in migrant care workers acted as surrogate caregivers, enabling family members to alleviate their daily caregiving burden, improve their emotional well-being, and sustain their personal lives. However, family caregivers faced several challenges, including resistance from dementia-affected relatives toward migrant workers, difficulties in recruiting care workers amidst fluctuating external conditions, and instances of migrant care workers displaying irresponsibility or lacking essential knowledge and skills related to dementia care. Notably, as family caregivers’ understanding of dementia evolved, they recognized the critical need to utilize public long-term care services to bolster the dementia-related knowledge and skills of their migrant employees. The study suggests implementing additional dementia-specific training programs tailored for both family caregivers and live-in migrant care workers in Taiwan. Such initiatives would enhance caregiving knowledge and competencies, ultimately improving the quality of life for both caregivers and care recipients.

**Key Points**

- Importance of Live-in Migrant Care Workers: There is a recognized dependence on live-in migrant care workers in Taiwan’s home-based eldercare, particularly due to demographic shifts and an increasing elderly population requiring support.
- Challenges in Dementia Care: Previous research indicates that family caregivers often face significant challenges, including emotional strain and difficulties in managing the care needs of relatives with dementia, which can be compounded when involving migrant workers.
- Qualitative Insights on Family Caregivers: This study fills a gap in qualitative research by providing in-depth insights into the motivations, benefits, and challenges faced by family caregivers in Taiwan when employing live-in migrant care workers specifically for dementia care.
- Evaluative Process for Hiring Migrant Workers: The research highlights the evaluative process that Taiwanese family caregivers undertake when deciding to hire migrant workers, emphasizing the consideration of personal circumstances and available resources in this context.
- Need for Enhanced Training Programs: The study suggests a critical need for implementing dementia-specific training programs for both family caregivers and live-in migrant care workers, aimed at improving caregiving knowledge and competences, which would ultimately enhance the quality of care and life for both caregivers and those they support.

## Introduction

As the global population ages, the number of individuals with dementia continues to rise annually. In 2018, Taiwan officially entered the stage of an aging society. According to the latest survey commissioned by the Ministry of Health and Welfare and conducted by the National Health Research Institutes, it is estimated that the prevalence of dementia among people aged 65 and older in Taiwan is about 8%.^1^ Dementia refers to a set of symptoms, including memory decline and deterioration in cognitive functions such as language, spatial awareness, calculation, and judgment, eventually leading to a loss of independent living abilities.^2^ Global data indicate that most individuals with dementia live at home, with family members as the primary caregivers.^3^ However, at any stage of dementia, individuals may exhibit Behavioral and Psychological Symptoms of Dementia (BPSD), such as agitation, confusion, depression, hallucinations, delusions, and anxiety.^4^ International research shows that behavior problems resulting from dementia are often a major source of stress for family caregivers, and that caregivers of individuals with dementia generally experience a lower quality of life compared to caregivers of older adults without dementia.^5^

For individuals with dementia and their families, especially during the mild to moderate stages, approximately corresponding to CDR 0.5 to CDR 2—remaining at home is often seen as the ideal living and caregiving environment.^6^ International dementia care research increasingly emphasizes that the quality of life of people with dementia depends significantly on meeting the needs and enhancing the quality of life of family caregivers.^5^ A systematic literature review on the needs of family caregivers of individuals with dementia categorized these needs into 2 main aspects^7^: first, receiving support and assistance to continue caregiving, which may require external resources such as formal services (eg, government-subsidized long-term care services like day care centers and respite services) and informal support (eg, assistance from family members); second, maintaining a balance between caregiver duties and personal needs, which may involve prioritizing one’s own health, maintaining existing lifestyles, and taking timely breaks. It is important to note that caregivers’ various needs are interrelated and are also influenced by the responses of individuals with dementia. For instance, caregivers might not use formal support services such as respite care because of perceived inadequacies in quality, flexibility, and convenience,^8^ or individuals with dementia may refuse to attend day care centers, leading families to hire migrant care workers as an alternative to maintain home-based care.^9^ Compared to Western countries, Asian societies—due to cultural and ethical beliefs such as Confucian filial piety—have a higher proportion of adult children caring for older relatives with dementia, and caregiving responsibilities are often shared among family members.^10^ However, in Taiwan, Singapore, and Hong Kong, many families hire migrant care workers to care for older relatives with dementia at home, mainly because adult children often have full-time jobs, childcare responsibilities, or do not co-reside with their parents.^11^

## Coping Strategies in Dementia Care

As mentioned earlier, to manage the demanding caregiving responsibilities while also attending to their own needs, family caregivers may seek various coping strategies, such as using respite services, securing time for themselves, turning to religious support, or seeking external help.^12,13^ In caregiving research, the coping model developed by Lazarus and Folkman has been widely used to explore how family caregivers respond to caregiving stress.^14^ “Coping” refers to caregivers’ evaluations of stressful events and available resources, and the strategies they adopt to manage or mitigate the stress, continually adjusting their approaches based on experience. Coping strategies are generally classified into 2 types: emotion-focused coping, which aims to regulate emotional responses, and problem-focused coping, which targets the source of stress through action and support-seeking. Later extensions of the model also identified diversion-focused coping, in which caregivers temporarily set aside caregiving duties to meet personal needs.^15^ In Singapore, where more than half of dementia families hire foreign domestic workers, Basnyat and Chang’s study highlighted that employing migrant care workers supported all 3 coping strategies by reducing daily caregiving burdens, improving emotional well-being, and allowing family caregivers to reclaim personal time.^16^

## Rationale of the Study

A 2011 survey in Taiwan showed that among 929 individuals with dementia, 93.8% lived at home, with 55.1% being cared for entirely by family members and 30.7% by migrant care workers.^15^ Despite the introduction of migrant labor since 1992, research specifically focusing on migrant care workers providing home-based dementia care in Taiwan remains limited. This may be due to the social stigma surrounding dementia^2^ or the sensitivity of discussing home care issues, which involve personal and family privacy. Consequently, qualitative studies exploring family caregivers’ experiences employing migrant care workers for dementia care are scarce. Few domestic studies have used in-depth interviews to explore migrant care worker employment in eldercare.^17,18^ Although some participants included individuals with dementia, the primary focus was not dementia caregiving, thus hindering a detailed exploration of the differences between caring for individuals with dementia and for those with general disabilities. Moreover, before 2020, Taiwanese policies limited LTC service access for families who had hired migrant care workers, discouraging concurrent use of government-funded LTC resources.^11^ As a result, past studies often emphasized that many caregivers hired migrant care workers as an alternative because “LTC services were insufficient” or due to a lack of knowledge about LTC options.^11,15,19,20^ Relatively few studies have considered whether hiring migrant care workers serves as an effective caregiving alternative from a coping perspective, or how individuals with dementia themselves react to care provided by migrant workers or LTC services.

## The Landscape of Dementia and Migrant Care in Taiwan

In response to global dementia policy trends, Taiwan published the “Dementia Prevention and Care Policy Framework (2014–2016)” in 2013, which was incorporated into the Long-Term Care 2.0 plan, expanding LTC services to individuals aged 50 and older with dementia.^21^ In 2018, the “Dementia Prevention and Care Policy Framework and Action Plan 2.0” was introduced, aiming to improve quality of life for people with dementia and their caregivers through support services, caregiver training, and consultation.^21^ Consequently, family caregivers now have more opportunities to acquire dementia-related knowledge and skills, enabling them to assist individuals with dementia in maintaining abilities and slowing decline.

In early 2020, the COVID-19 pandemic severely disrupted the inflow of migrant care workers into Taiwan, while Indonesia’s “zero-cost” policy reforms shifted placement costs onto employers. As Indonesia supplies 75% of Taiwan’s migrant care workers, this policy led to resistance among Taiwanese employers, hiring delays, and wage increases.^22^ Although Taiwan subsequently negotiated policy exemptions and adjusted migrant care worker’s wages, domestic worker shortages persisted. Unlike industrial workers, domestic workers are not covered by labor standards regulations, resulting in lower wages, fewer rest days, and limited employment mobility, driving many to seek better-paid factory jobs.^23^ By March 2022, the situation had worsened when the Indonesian government suspended the verification of newly hired caregivers, resulting in a backlog of 64 000 employers waiting to recruit migrant care workers. Following multiple rounds of negotiation between the Taiwanese and Indonesian governments, Taiwan agreed to raise the monthly salary for domestic workers from €470 EUR to €554 EUR. In return, Indonesia exempted Taiwan from the zero-cost policy and reopened the recruitment of caregivers to Taiwan.^24^

Today, many developed countries increasingly depend on migrant care workers for dementia and elder care.^24,25^ Some, such as Japan and Finland, restrict migrant labor to institutions only, while others, like Taiwan, allow migrant workers to provide home-based care.^26,27^ In Taiwan, recent changes in policy and broader environmental factors, the procedures and economic costs associated with hiring migrant care workers for home care of elderly individuals with disabilities or dementia have increased. As the pandemic situation has eased, the number of hired migrant care workers has begun to rise again (see Table 1). Despite these trends, Taiwan’s situation remains under-researched, particularly regarding how pandemic disruptions and LTC reforms have reshaped family caregiving decisions and experiences.

**Table 1.** Number of Migrant Care Worker’s Introduced in Taiwan in Recent Years.

Year end	Total migrant care workers	Caregivers in long-term care institutions	Home care services
End of 2019	259 660	15 281	244 379
End of 2020	250 188	15 712	234 476
End of 2021	225 423	15 215	210 208
End of 2022	220 337	16 693	203 644
End of July 2023	226 926	17 369	209 557

*Source.* Ministry of Labor Statistics Inquiry Website, retrieved from https://statdb.mol.gov.tw/statiscla/webMain.aspx?sys=100&kind=10&type=1&funid=wqrymenu2&cparm1=wq14&rdm=I4y9dcIi.

LTC 2.0 now permits families hiring migrant care workers to also access government-subsidized LTC services. Thus, caregivers have more flexible options than simply choosing between hiring migrant workers or using formal LTC services. Considering these policy expansions and resource developments, it is important to examine why many dementia caregivers in Taiwan still rely heavily on migrant care workers. Do migrant care workers fully meet the needs of dementia families, or is additional LTC support necessary? This study aims to explore, through qualitative interviews with family caregivers, whether hiring migrant care workers effectively alleviates caregiving stress, what challenges caregivers encounter, and how migrant care worker employment integrates (or fails to integrate) with Taiwan’s LTC system.

## Materials and Methods

### Study Design

A qualitative research methodology was employed to address the aims of the study. Each participant was interviewed 3 times—prior to and following the training program—and the interview transcripts were analyzed using thematic analysis. The caregivers participated in a 6-session training program totaling 24 h, titled *“Program for Coping with Behavioral and Psychological Symptoms of Dementia (PCBPSD).”*

### Participants

Participants were recruited through the neurology outpatient clinic of a regional hospital in southern Taiwan. The study focused on 4 family caregivers, aged between 52 and 63 years, who had either currently or previously employed migrant care workers to assist in the care of relatives with dementia. All participants were adult children of individuals with dementia. To be eligible, caregivers had to be the primary family caregivers, with at least 1 year of caregiving experience for a family member with dementia living in the same household or nearby. Basic demographic and caregiving-related information about the participants and their family members with dementia is provided in Table 2.

**Table 2.** Basic Information of Respondents and Their Family Members with Dementia.

Respondent	Age/Gender	Relationship to care recipients/family support	Care recipient’s age	Duration of caregiving (years)/employment situation	Care situation
A1	54/M	Son/Partial	79	2/Part-time	Requires assistance with activities of daily living; initially resisted caregiver support but later accepted care from migrant care workers.
A2	52/M	Son/Non	85	2/Unemployed	Requires assistance with activities of daily living; resists care from outsiders and may physically react against migrant care workers; has available support at dementia care facilities.
A3	63/F	Daughter/Non	85	3/Housewife	Initially received assistance with activities of daily living from migrant care workers, but later, the caregivers escaped.
A4	53/F	Daughter/Non	82	9/Full-time	Highly dependent on migrant care workers for assistance with activities of daily living.

### Data Collection

Data collection was conducted between April and August 2022. A total of 8 family caregivers were initially recruited, from which 4 were selected for inclusion based on their experience with employing migrant care workers. To understand their caregiving experiences, the researcher conducted 3 individual, in-depth interviews with each caregiver: 1 before the training program and 2 following its completion. A semi-structured interview format was used to foster a comfortable environment in which participants could freely express their caregiving experiences. Guided open-ended questions allowed respondents to articulate their thoughts using their own language and vocabulary, enabling a richer and more nuanced understanding of their perspectives. This approach supported both verbal and non-verbal communication, promoting a deeper comprehension of respondents’ experiences.^28^

Each interview followed a pre-designed outline developed by the researcher and research team, covering key areas such as daily caregiving routines and challenges, interactions with individuals with dementia, use of long-term care services or migrant care workers, and perceived changes in caregiving experiences after the training program. All interviews were audio-recorded with participants’ consent and transcribed verbatim.

### Data Extraction and Analysis

The analysis focused on the experiences of the 4 family caregivers who had employed migrant care workers. The interview data analyzed included transcripts from the 3 interviews conducted over the 5-month study period: 1 prior to the training and 2 follow-up interviews conducted at 2 months post-training. Participants reflected not only on their caregiving experiences but also on how the training program influenced their views and decisions regarding the employment of migrant care workers. Although the sample size was small, data collection and analysis continued until thematic saturation was reached—meaning no new themes emerged in the final stages of analysis—thereby ensuring analytical depth and credibility.^29-31^

Thematic analysis was employed as the analytical approach.^32^ Transcripts were read multiple times by the researcher and research team members, who used an inductive method to identify and refine themes emerging from the data. Key themes included: employment of migrant care workers to meet the needs of in-home care for individuals with dementia, advantages of employing migrant care workers for caring for family members with dementia, and issues or concerns arising from employing migrant care workers. Themes and sub-themes were discussed and refined collaboratively to ensure consistency and depth in analysis.

To ensure the rigor of the study, 4 criteria proposed by Lincoln and Guba were followed^29^:

1. **Credibility:** The principal investigator developed sustained engagement with the participants over 6 months through the training sessions and interviews, enhancing the authenticity of the data.
2. **Transferability:** Cross-case comparisons revealed commonalities among participants’ experiences, which align with findings in similar contexts, suggesting that the data reflect broader caregiving realities.
3. **Dependability:** After establishing a consistent coding system through collaborative discussions, each research team member independently coded the transcripts. Results were then compared to ensure coding consistency and reliability.
4. **Confirmability:** Multiple readings of the transcripts and reflective discussions helped mitigate subjective bias. Thematic analysis involved rigorous cross-checking and dialog among the team to minimize interpretative discrepancies.

This study aimed to present the lived experiences of family caregivers in employing migrant care workers, and the resulting impact on both family caregivers and individuals with dementia.

### Research Ethics

This study was approved by the Research Ethics Committee of the National Health Research Institutes on March 17, 2022 (IRB number: EC1110102). The research team adopted 3 steps to safeguard the rights and welfare of research participants. First, prior to recruitment, potential participants were provided with detailed information regarding the research objectives, the content of the training course, the interview process, the mechanism for withdrawing from the study and interviews, as well as the handling and use of interview data. Participants were then asked to give their consent by signing a “Research Participation Consent Form.” Second, during each interview, the researchers briefly reiterated the purpose of the study, confirmed the respondents’ willingness to continue participating, and remained attentive to the circumstances of the respondents and their family members with dementia. Interview questions and lengths were flexibly adjusted, especially considering that caregivers might need to conclude interviews early due to unforeseen situations concerning their family members. Finally, to ensure the privacy and confidentiality of research participants, all research data were analyzed and reported using codes and anonymous identifiers.

## Results

From the interview data, 3 main themes and their corresponding subthemes were identified (see Table 3). The following sections elaborate on these findings based on the data collected.

**Table 3.** The Main Themes and Subthemes.

Themes	Subthemes
Employment of migrant care workers to meet the needs of in-home care for individuals with dementia	(1-1) Lack of family support
	(1-2) Full-time employment or other caregiving responsibilities
	(1-3) Reluctance of individuals with dementia to attend day care
	(1-4) Need to balance personal life
Advantages of employing migrant care workers for caring for family members with dementia	(2-1) Alleviation of caregiving burdens and stress
	(2-2) Migrant care workers provide continuous 24-h care and immediate support
	(2-3) Migrant care workers provide one-on-one care, facilitating familiarity
	(2-4) Ability to balance personal life
Issues or concerns arising from employing migrant care workers	(3-1) Resistance from individuals with dementia
	(3-2) Adjustment between migrant care workers and employer family require times
	(3-3) Uncertainty regarding the retention of migrant care workers
	(3-4) Irresponsibility migrant care workers
	(3-5) Migrant care workers’ lack of knowledge and skills in caring for individuals with dementia

### Theme 1. Employment of Migrant Care Workers to Meet the Needs of In-Home Care for Individuals With Dementia

All 4 interviewed family caregivers resided with or near their relatives with dementia and identified themselves as the primary caregivers. Both caregivers and care recipients preferred home-based living; however, progressive cognitive and physical declines often led to wandering and disorientation in individuals with dementia, necessitating continuous supervision. The resulting burden of 24-h care contributed to the decision to employ migrant care workers. The caregivers shared a common rationale: maintaining their relatives’ quality of life at home while alleviating the caregiving burden on themselves.

#### (1-1) Lack of Family Support

Respondents consistently reported a lack of caregiving support from other family members, who cited geographical distance, personal responsibilities, or work obligations. All 4 primary caregivers were adult children of the individuals with dementia. Despite cultural values related to filial piety, the decision to employ migrant care workers was driven by personal circumstances. For instance, A1 (Age 54, Son), who had inherited most of his parents’ assets, perceived this as contributing to his siblings’ reluctance to assist caregiving. Reflecting traditional gender expectations and inheritance norms, A1 remarked:

*I cannot say that my sisters should also share the burden because I have already received more than they have.*

A1 shared this that his parents might view their daughters, after marriage, “*as belonging to their husband’s families*”—an attitude influencing his decision to assume full responsibility by hiring migrant care workers. This aligns with traditional Chinese family structure, where caregiving often defaults to sons, especially those who inherit family assets.

#### (1-2) Full-Time Employment or Other Caregiving Responsibilities

Participant A3 (Age 63, Daughter), who has a full-time job, and A4 (Age 53, Daughter) face additional caregiving challenges– 1 simultaneously cared for a child with disabilities, while the other held a full-time job. Both found it necessary to hire migrant care workers for continuous supports. A4 recounted a critical incident:

Why do I hire migrant care workers? Because I work every day, and her (my mother’s) blood sugar is not well controlled. There was once when I was at work, she was sleeping in a chair, her eyes were open, and she was foaming at the mouth.

#### (1-3) Reluctance of Individuals With Dementia to Attend Day Care Center

Caregivers expressed a preference for their relatives to engage in social activities at Day Care Centers; however, resistance from individuals with dementia often limited this option. A1 (Age 54, Son) observed that his mother exhibited a poor mood after attending Day Care and thus withdraw her. Similarly, A4’s mother (Age 53, Daughter) declined to return after 3 days, citing discomfort among the other attendees:

She felt out of place with the other elderly individuals. I went to check in on her, and my mother was actually the oldest *one* there, but she appeared the youngest. She believed that she did not fit in with those older people.

#### (1-4) Need to Balance Personal Life

The unpredictability and duration of dementia care significantly impacted caregivers’ personal and professional lives. A2 (Age 52, Son), paused his overseas employment due to COVID-19, and his mother’s diagnosis, but expressed a desire to return to work:

As long as I can manage financially, I can temporarily set my job aside, but in the long term, I still need to find new employment. . .I need a migrant care worker to help to care of my mother.

A1 (Age 54, Son) also noted full-time caregiving initially disrupted his family life, but came to recognize that hired care could preserve family relationships:

[I shouldn’t think that] I am being tied down by my parents. . .I need someone to care for them, but does it have to be the children providing that care directly? For instance, hiring migrant care workers. . .could lead to a more harmonious parent-child relationship!

### Theme 2. Advantages of Employing Migrant Care Workers for Caring for Family Members With Dementia

Family caregivers who currently or previously employed migrant care workers reported several benefits associated with this caregiving management. The presence of migrant care workers helped reduce both physical and psychological stress, providing continuous and individualized care, and enable caregivers to balance competing personal responsibilities.

#### (2-1) Alleviation of Caregiving Burdens and Stress

All 4 caregivers expressed that migrant care workers significantly alleviated their caregiving load. Initially, A1 (Age 54, Son) attempted to use paid Long-term home care service to assist with his mother’s hygiene’s needs; however, his mother exhibited strong resistance and even became aggressive toward the caregiver. This led A1’s father, despite having a chronic illness, to take over bathing responsibilities. As his father’s health declined, A1 engaged in repeated discussions with both parents, eventually gaining their consent to hire a migrant care worker. The support provided by the migrant care worker substantially eased the family’s caregiving burden.

Similarly, A3 (Age 63, Daughter), who was the sole caregiver co-residing with her mother, noted that the migrant care worker’s presence allowed her to share the emotional and physical weights of caregiving. A4 (Age 53, Daughter), who had employed family member caring for his mother with dementia, has employed a migrant care worker for 9 years, shared that her mother frequently displayed problematic behaviors such as nighttime agitation, which severely affected A4′s work and well-being. A4 stated:

If I did not have a migrant care worker today, and it was truly me [providing care] every day, I don’t think I could maintain such an open mindset!

A4 also emphasized that the present of migrant care worker provided her with emotional capacity to personally care for her mother during the caregiver’s days off, without the needs to utilize respite services offered by government Long-term care programs:

This is a time for me to be alone with her, but there is someone (the migrant care worker) helping me. I feel an extreme need for this kind of service and assistance.

#### (2-2) Migrant Care Workers Provide Continuous 24-Hour Care and Immediate Support

Unlike time-limited services provided by paid caregivers under government’s Long-term care programs, migrant care workers are available around the clock. This constant presence is particularly valuable for families with complex caregiving needs. For instance, A3 (Age 63, Daughter), who was simultaneously responsible for a son with disabilities, relied on a migrant care worker to provide a full-time care for her mother, enabling her to manage both roles more effectively. A3 also emphasized the importance of immediate physical assistance:

I am very fortunate to have a migrant care worker for tasks that require hands-on involvement– they really work hard. They are unable to work elsewhere and, there must be someone dedicated to providing care right next to such an elderly individual.

#### (2-3) Migrant Care Workers Provide One-on-One Care, Facilitating Familiarity

Several participants noted that individuals with dementia have difficulty adjusting to unfamiliar people entering their homes, especially when caregivers change frequently. A1 (Age 54, Son) explained:

[Paid caregivers from Long-term care services] can pose challenges for individuals with dementia because they need familiar people to care for them, but caregivers frequently change. The elderly may exhibit resistance due to this unfamiliarity.

Hiring a migrant care worker helped reduce this resistance and built emotional rapport over time. A1 expressed satisfaction upon witnessing a meaningful interaction between his mother and the migrant care worker:

Yesterday, I took my mother out, and the migrant care worker accompanied us to the clinic to pick up medication. I even saw my mother in the examination room telling the caregiver, 'I am very grateful that you are here to take care of me.’ Witnessing this moment was quite comforting for me.

#### (2-4) Ability to Balance Personal Life

A recurring theme among the participants was the desire to regain a sense of autonomy and personal balance. With no other family members available to help and competing responsibilities such as full-time employment, family caregivers found that hiring migrant care workers provided peace of mind and allowed them to reclaim time for themselves. As A1 reflected:

I feel that I can now be the master of my time management, because in the past, without the help of migrant care worker, the conditions of my parents were sometimes unstable, which made it difficult for me to engage in any activities.

### Theme 3. Issues or Concerns Arising From Employing Migrant Care Workers

While migrant care workers offer essential support for home-based dementia care, family caregivers also reported multiple challenges in their employment, including care recipient resistance, communication barriers, caregiver reliability, and inadequate dementia-specific training.

#### (3-1) Resistance From Individuals With Dementia

Despite the intention to reduce caregiving burden, individuals with dementia sometimes resisted the presence of migrant care workers, leading to behavior problems and tensions. For example, A2 (Age 52, Son) described how his mother became physically aggressive toward caregivers, resulting in the replacement of 4 migrant care workers within 2 years. As A2 reflected:

[My mother] initially expressed her refusal by simply swinging her arms, but as her condition worsened, when the migrant care worker approached her, she would strike at her. If the caregiver tried to move away, she would even chase after her. Although she is sitting in a wheelchair, her hand would reach out to hit her—that type of situation. She clearly expresses that she does not like the migrant care workers.

Due to the mother’s resistance and continued aggressive behavior toward the migrant care workers, A2 has already gone through 4 different caregivers within 2 years. Nevertheless, A2 still hopes to retain a migrant care worker because having someone to assist with caring for the mother makes “*life feel at least a bit easier.*” Similarly, A3 (Age 63, Daughter) noted that her mother was dissatisfied with her migrant care worker’s behavior, perceiving her as inattentive and wasteful, particularly critical of her use of a mobile phone and arguing back. Resistance was often rooted in the older adults’ sense of autonomy, frugality, and discomfort with unfamiliar individuals.

#### (3-2) Adjustment Between Migrant Care Workers and Employer Families Requires Time

Mutual adjustment between migrant care workers and employers’ families emerged as an recurring issue. A2 (Age 52, Son) observed language barriers and cultural misunderstandings contributed to misinterpretation of migrant care workers’ intentions of tone, causing distress for his mother. As A2 stated:

Originally, the migrant care worker was supposed to come to solve a problem, but there are also new adaptation issues that need to be addressed. Previously, when trying Long-term care services, if the caregiver changed, I had to teach them again; it’s the same with migrant care workers.

With regard to my mother’s emotional difficulties, caregivers must spend long periods of time accompanying and understanding her to be patient in their care. Otherwise, whenever a new person comes in, my mother is surprised by the new person, which leads to reactions beyond her expectations. In such a case, the caregiver may not last long and we must start over again.

In addition, migrant care worker unfamiliar with dementia care needed extensive on-the-job instructions from the families. A2 commented that each new caregivers—whether from Long-term care services or migrant care workers—required training from scratch, which was time-consuming and emotionally taxing. Recurring changes disrupted routines and negatively affected the person with dementia, reinforcing the need for continuity and preparation.

#### (3-3) Uncertainty Regarding the Retention of Migrant Care Workers

The retention of migrant care workers was further complicated by policy shifts and labor market fluctuations. The COVID-19 pandemic, along with changes in Taiwan’s migrant labor policies, significantly impacted the availability and stability of the migrant caregiving workforces. A2 (Age 52, Son) reported losing the migrant care worker due to a herniated disc and being unable to retain a potential replacement, who instead chose to pursue a higher-paying job in a factory. To retain his forth migrant care worker, A2 took on intimate caregiving task himself, such as bathing and relied on an agency to assist with communication. He remarked:

I would respect her decision if she wished to change jobs, but the high instability of the migrant care workers made my burden even worse.

Even when a migrant care worker was deemed suitable, families faced uncertain about their long-term availability. A1 (Age 54, Son) expressed concerns about migrant care workers’ legal ability to switch employers freely, stating:

The biggest worry right now is how long the caregiver can stay?

#### (3-4) Irresponsible Migrant Care Workers

Some participants expressed frustrations with migrant care workers’ perceived lack of professionalism or commitment. A3 (Age 63, Daughter) reported that her mother complained frequently about migrant care worker’s attitude, ultimately leading to the caregiver leaving the position. However, due to A3’s dual caregiving responsibilities, A3 explained to the mother, “*That’s how most foreign workers are nowadays*,” urging her mother to “*bear with it a little.*” Ultimately, the caregiver absconded. A4 (Age 53, Daughter), who had employed migrant care workers for nearly a decade, disclosed that her current migrant care worker had repeatedly stolen money. Despite these issues A4 chose to retain the migrant care worker to avoid the effort of retraining a new one, even considering nursing home care as an alternative. A4 remarked:

I don’t want to change caregivers. . . because then I would have to train another *one* and go through the process of adjusting all over again. . . It’s something that requires working through. . . I might consider a long-term care [facility] instead.

These experiences illustrate the emotional and logistical burdens associated with migrant care worker turnover and misconduct.

#### (3-5) Migrant Care Workers’ Lack of Knowledge and Skills in Caring for Individuals With Dementia

A consistent concern was the lack of dementia-specific skills among migrant care workers. Several family members observed that migrant care workers, while competent in basic tasks, often failed to encourage autonomy in person with dementia, potentially accelerating functional decline. A4 (Age 53, Daughter) reflected that her mother had become overly dependent, regressing in her ability to perform basic tasks. As A4 reported: *“My mother gradually became reliant. . .[then later] too reliant, which led to a regression in her habits and abilities.*” In contrast, A1 (Age 54, Son) who had completed a dementia care training course, emphasized the importance of promoting remaining abilities. He collaborated with a local social worker and an occupational therapist– both assigned through Long-term care services—and was assisted by an Indonesian interpreter to help the migrant care worker facilitate simple household activities that had previously given his mother a sense of purpose. As A1 stated:

My main goal was to ensure the migrant care worker understood that beyond meeting daily care needs, there are many activities to fill the time. They offered several suggestions; my mother used to do simple chores, like sweeping and vegetable picking, so they encouraged doing those together—because she isn’t inclined to do it on her own anymore.

Likewise, A2 (Age 52, Son) invited a dementia care instructor to provide a in-home guidance to the migrant care worker, aiming to establish a structured daily routine. Based on these experiences, both A1 and A2 recommended the development of training programs or multilingual manuals tailored to migrant care workers’ needs to improve the quality of dementia care.

## Discussion

For many family caregivers of individuals with dementia, caregiving is physically, psychologically, and financially a demanding process filled with uncertainty. In Taiwan, the majority of people with dementia living at home during the mild to moderate stages—approximately corresponding to CDR 0.5 to CDR 2—primarily cared for by family members. While this is aligned with international dementia care policies that emphasize home- and community-based care, the trend is also influenced by the shortage of professional dementia care institutions, cultural resistance to institutionalization, and the enduring values of Confucian filial piety. Although family caregivers often wish to care for their loved ones at home, practical limitations such as employment obligations, family dynamics, and the progressive nature of dementia often lead them to hire migrant care workers as a practical solution.

This study found that employing migrant care workers can serve as a viable caregiving alternative. However, it also raises questions about how well Taiwan’s current long-term care services (LTC) can support both the caregivers and the care recipients in these arrangements. The discussion below is organized into 3 main areas: (I) employment of migrant care workers as an alternative caregiving solution, (II) issues and concerns faced after hiring migrant care workers, and (III) the potential role of long-term care services in addressing caregiving gaps.

### Employment of Migrant Care Workers as an Alternative Caregiving Solution

These findings indicated that the decision to employ migrant care workers is not a simple delegation of filial piety but a pragmatic response to complex caregiving challenges. Family caregivers assess their personal capacity, financial resources, availability of other family support, and the willingness of care recipients to use Day Care services before making this decision. For example, A1 (Age 54, Son) justified his decision by acknowledging that although he inherited more family assets, he did not wish to sacrifice his own quality of life entirely. Hiring a migrant care worker, in this context, was a way to preserve family harmony and personal well-being.

Participants in this study described how migrant care workers helped alleviate the physical and emotional strains of caregiving. By residing in the same household, these migrant care workers provided constant supervision and companionship, enabling family caregivers to regain control of their time and attend to other responsibilities. These findings are consistent with prior studies that have examined the reasons caregivers opt to hire migrant care workers or use long-term care services.^17,21^

While much of the existing literature focuses on the perspectives of family caregivers, fewer studies explore the views of individuals with dementia themselves.^11^ This study contributes to that gap by highlighting how individuals with dementia, even in the early or moderate stages, can influence caregiving arrangements through their preferences or resistance behaviors. For example, some respondents shared that their relatives refused to attend Day Care due to feelings of unfamiliarity or perceived incompatibility with other attendees. As a result, families viewed hiring migrant care workers as a more acceptable and personalized alternative. The findings of this study are consistent with international research showing that people living with dementia can still clearly express dissatisfaction with certain caregiving approaches, or communicate their preferences or needs through problematic behaviors.^12,31^ Such expressions often prompt families to seek alternative forms of care, including the employment of migrant care workers.^9,11^

### Issues and Concerns Faced After Hiring Migrant Care Workers

Despite their utility, the employment of migrant care workers introduced new challenges. Respondents noted frequent issues such as communication difficulties due to language barriers, mismatches in cultural expectations, and a lack of dementia-specific care knowledge. Several caregivers reported that their relatives with dementia showed resistance to the caregivers, perceiving them as strangers or intruders. In A2’s (Age 52, Son) case, this led to aggressive behaviors or the need to replace the migrant care workers multiple times. Additionally, some family caregivers encountered problems related to the conduct and professionalism of migrant care workers. Reports included excessive phone use, arguments, attitudes, or even theft. These findings are also consistent with previous literature showing migrant care workers exhibiting irresponsibility, absconding, or stealing.^21,22^ These incidents often increased caregivers’ stress and raised concerns about safety and trust. At the same time, the participants expressed reluctance to replace a caregiver due to the burden of retraining a new one.

The lack of standardized dementia care training for migrant care workers is another significant concern. Although migrant care workers from countries like Indonesia undergo pre-departure training, this often lacks content specific to dementia care.^32^ Upon arrival in Taiwan, their exposure to local dialects such as Minnan or Hakka further complicates communication. Compared to institutional caregivers who may receive on-the-job training tailored to specific clinical scenarios, particularly in managing the behavioral challenges posed by individuals with dementia,^32^ in-home migrant care workers typically face dementia-related care challenges alone, with little access to professional support. These findings align with research in Taiwan and other Asian contexts that emphasize the emotional and professional burden placed on under-trained migrant care workers.^18,33,34^

Recent global events, particularly the COVID-19 pandemic and changes in labor export policies, have further disrupted the hiring and retention of migrant care workers.^26^ Family caregivers shared concerns about workforce instability, frequent turnover, and the unpredictability of whether migrant care workers would remain in their roles long-term. Some reported that potential migrant care workers opted for better-paying jobs in manufacturing or left their position unexpectedly, creating significant stress and care gaps.

### The Potential Role of Long-Term Care Services in Addressing Caregiving Gaps

Taiwan’s Long-Term Care Program 2.0 provides an important support structure for families that employ migrant care workers. Respondents in this study described how they accessed home-based services, arranged Day Care visits for their relatives, or participated in caregiving training programs to strengthen their own knowledge of dementia. Participation in programs such as the *“Program for Coping with Behavioral and Psychological Symptoms of Dementia (PCBPSD)”* helped caregivers gain practical skills and a deeper understanding of how to guide and communicate with migrant care workers more effectively. Importantly, the participants noted that early access to such training might have helped prevent excessive dependence on caregivers, which they believed contributed to a decline in their relatives’ remaining abilities. By becoming more knowledgeable themselves, migrant care workers were better equipped to maintain an active role in the caregiving process and avoid complete delegation to undertrained care workers.

While some respondents had positive experiences with integrating long-term care services alongside migrant caregiving, data also revealed that many families do not take advantage of the available resources. A 2021 telephone survey by Wu and Lin found that only 37.8% of employers of migrant care workers were interested in using home care services, and 42% believed they did not need long-term care support.^21^ These findings pointed to a gap in awareness and uptake. Strengthening policy advocacy and outreach to family caregivers is essential to improve the quality of dementia care at home. Since family members act as gatekeepers for both the training and funding of migrant care workers, equipping them with the right knowledge is a critical step toward integrating long-term care services more fully with in-home migrant support.

## Strengths and Limitations

This study presents in-depth qualitative insights from 4 family caregivers of individuals with dementia who are currently or have previously employed migrant care workers in Taiwan, and thematic saturation was achieved following established qualitative research principles.^28^ While the small sample size limits the generalizability of the findings, the study achieved thematic saturation, a key criterion for rigor in qualitative research. The consistency and recurrence of themes across interviews suggest that the data adequately capture the core dynamics experienced by their caregiver group.

The strengths of this study lie in its rich, contextual understanding of caregiving decisions and dilemmas, providing nuanced accounts that reflect both cultural and structural factors influencing care arrangements. The qualitative design enabled a deeper exploration of personal experiences, values, and evolving strategies in navigating home-based dementia care. Nonetheless, the study’s limitations must be acknowledged. The findings may not represent the full range of experiences of all families employing migrant care workers, particularly in different regions or socioeconomic contexts. In addition, while the focus on family caregivers offers valuable perspectives, future research should include the voices of care recipients and migrant care workers themselves to better understand their interactions and roles within the caregiving triad.

### Recommendations

Based on the findings and challenges discussed above, we offer the following recommendations to improve dementia care for families employing migrant care workers:

1. **Enhance Training for Migrant Care Workers:** Government and community organizations should develop standardized, dementia-specific training programs that are culturally and linguistically appropriate. These materials should be offered in the migrant care workers’ native languages (eg, Indonesian, Vietnamese) and include content on behavioral management and communication. In addition, Mandarin language proficiency standards (eg, CEFR A1 or A2 levels) should be established for entry-level migrant care workers. Accessible language learning resources should be made available to help migrant care workers meet these requirements and improve communication with care recipients.
2. **Promote Post-Diagnosis Education for Family Caregivers and People Living with Dementia:** Training programs like the *“Program for Coping with Behavioral and Psychological Symptoms of Dementia (PCBPSD)”* should be made more widely available to family caregivers at the time of diagnosis. This would help them guide migrant care workers more effectively and delay dependency in care recipients. Also, employers of migrant care workers themselves are required to attend training programs like the PCBPSD course. In addition, they should provide paid on-leave for migrant care workers to attend the training programs.
3. **Integrate Long-Term Care Services with In-Home Migrant Support:** Families employing migrant care workers should be encouraged to use available LTC 2.0 services such as home care guidance, psychological counseling, and respite care. Coordinated service models should ensure that formal and informal care providers work collaboratively.
4. **Improve Retention of Migrant Care Workers:** Address labor market disparities that lead to high turnover by offering incentives for migrant care workers to remain in eldercare positions. Streamline administrative processes and improve job stability for migrant care workers.
5. **Raise Awareness and Policy Engagement:** The post-diagnosis support network should be built on collaboration among medical, long-term care, and community-based providers. This approach aligns with global frameworks such as the WHO’s Global Action Plan on Dementia, the UK’s National Dementia Strategy, and Japan’s Orange Plan—all of which emphasize integrated, community-based, and multisectoral care systems. Drawing on these models can inform Taiwan’s effort to build a sustainable dementia pathway. Stronger advocacy is needed to ensure that dementia care remains a national priority.

These recommendations aim to support a more sustainable, integrated, and person-centered care system that enhances the well-being of both individuals with dementia and their caregivers.

## Conclusions

This descriptive and exploratory study aimed to gain an in-depth understanding of the reasons, perceived benefits, and concerns associated with the employment of migrant care workers by family caregivers of individuals with dementia in Taiwan. Through qualitative interviews, main findings emerged. First, regarding the reasons for hiring migrant care workers, caregivers sought to meet the intensive demands of in-home dementia care in the absence of adequate support from other family members. Their decision was shaped by their work obligations, additional caregiving responsibilities, and the need to maintain their own well-being. The decision-making process also involved an evaluation of personal resources and reinterpretations of filial piety within their sociocultural context. Second, in terms of the advantages, migrant care workers were described as essential supports in alleviating caregiving burdens, reducing emotional stress, and enabling family caregivers to return to work or manage their personal lives. This provision of continuous, immediate, and individualized care helped address practical, emotional, and diversionary caregiving needs. Third, concerns raised by caregivers centered on language barriers, inconsistent care quality, and the lack of dementia-specific skills among migrant care workers. Family caregivers also noted challenges related to care recipients’ resistance, the complexity of dementia care compared to general elder care, and policy-driven labor shortages. These challenges highlighted the need for better integration between family caregiving, migrant labor, and formal long-term care systems.

Overall, while hiring migrant care workers can offer practical solutions to dementia caregiving, the strategy must be regularly reassessed to account for evolving care needs, caregiver expectations, and broader policy shifts. As family caregivers become more informed about dementia and available resources, they recognize the importance of not fostering over-dependence on migrant care workers. Instead, they increasingly value using long-term care services—such as professional home visits and caregiver training—to support migrant care workers in sustaining the cognitive and functional abilities of individuals with dementia.
